# Long-term results of the iBP elbow prosthesis: beware of destructive metallosis!

**DOI:** 10.1186/s12891-019-2781-z

**Published:** 2019-09-06

**Authors:** Daniëlle Meijering, Alexander L. Boerboom, Fred Breukelman, Denise Eygendaal, Sjoerd K. Bulstra, Martin Stevens

**Affiliations:** 1 Department of Orthopedic Surgery, University of Groningen, University Medical Center Groningen, Groningen, the Netherlands; 2 Orthopedic Department, Wilhelmina Hospital, Assen, The Netherlands; 3 Department of Orthopedic Surgery, Amsterdam Medical Center, Amsterdam, The Netherlands; 4 Orthopedic Surgery Department, Amphia Hospital, Breda, the Netherlands

**Keywords:** iBP, Elbow prosthesis, Unlinked elbow prosthesis, arthroplasty, Long-term follow-up

## Abstract

**Background:**

The aim of this study was to review the long-term results of the instrumented Bone Preserving (iBP) elbow prosthesis.

**Methods:**

Thirty-one patients (10 M, 21F, 28-77 year) were retrospectively evaluated using the Oxford Elbow Score (OES), Disabilities of Arm, Shoulder and Hand Outcome Measure (DASH), Mayo Elbow Performance (MEPS), physical examination and standard radiographs. Kaplan-Meier survival analysis was used.

**Results:**

Thirty-seven primary iBPs have been placed in 31 patients between 2000 and 2007. Six patients (8 prostheses) had died, 10 elbows had been revised and three patients (4 prostheses) were lost to follow-up. Fourteen patients (15 prostheses) were available for follow-up. The main indication for surgery was rheumatoid arthritis. Mean follow-up was 11 years (8–15). Kaplan-Meier survival analysis showed a survival of 81% at 10 years after surgery. Main reason for revision was particle disease and loosening due to instability and malalignment. Eleven of 14 patients were satisfied, although radiographs showed radiolucencies in 11 patients.

**Conclusion:**

The iBP elbow prosthesis gives a survival rate of 81% 10 years after surgery with a progressive decline beyond 10 years. However, many patients have radiolucencies. Discrepancy between clinical signs and radiological results warrants structural follow-up, to assure quality of bone stock in case revision surgery is indicated.

The study was reviewed and approved by the Medical Ethical Committee of University Medical Center Groningen (METc2016/038).

**Level of evidence:**

Level IV, Case series.

## Background

Joint destruction due to inflammatory arthritis is still the main reason for a total elbow arthroplasty (TEA), although nowadays posttraumatic osteoarthritis is a more common indication for a replacement [1, 2]. As the prevalence of TEA is low compared to knee and hip arthroplasties, reports with long-term follow-up are rather scarce.

**Fig. 1:**
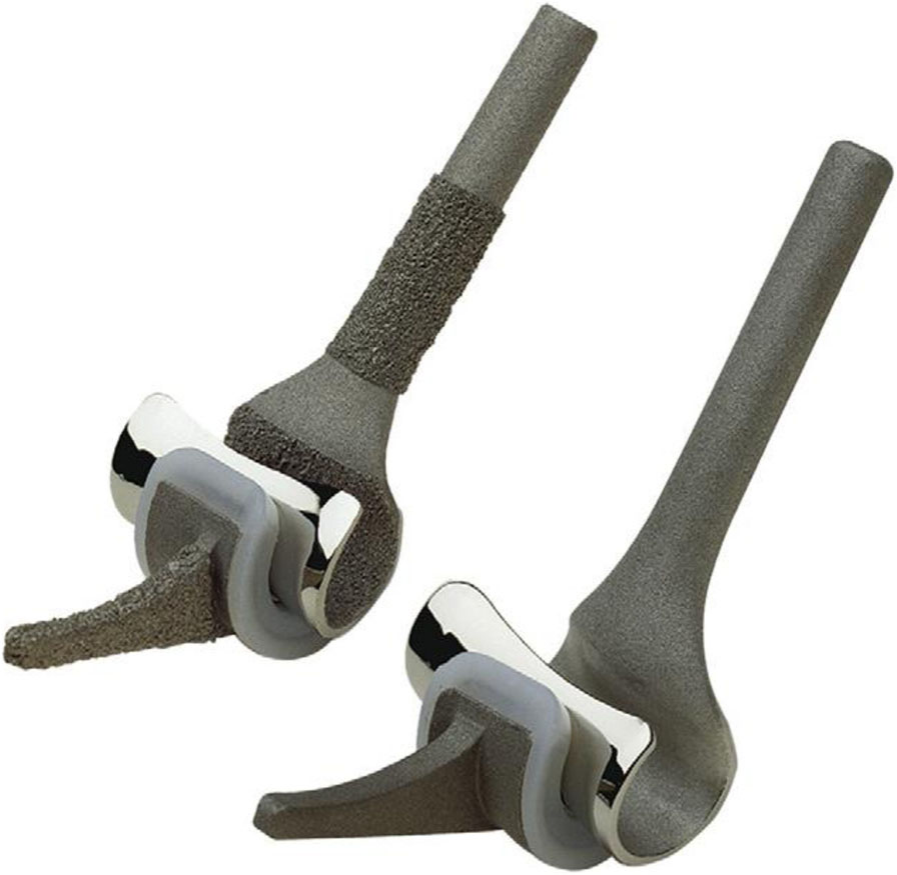
iBP uncemented (left) and cemented (right) ulnar and humeral components

Over the last 40 years there have been many improvements in the design of total elbow prostheses. In general, three types of prostheses are available: unlinked devices, linked devices and convertible devices, which can be used as either a linked or an unlinked system. The instrumented Bone Preserving (iBP) (Biomet, Warsaw, IN, USA) elbow prosthesis is an unlinked, ulnohumeral prosthesis, designed by Pooley. The iBP elbow prosthesis is a modification of the Kudo type 5, developed to preserve more bone stock (Fig. 1) [3]. A study with a mean follow-up of 49 months by Kleinlugtenbelt et al. [4] showed a discrepancy between clinical outcome and radiological signs. Patients scoring good-to-excellent on the Mayo Elbow Performance Score (MEPS) did show radiolucencies around their ulnar component, while patients with a poor MEPS score did not. As a result of this discrepancy, progressive radiolucency can occur without any clinical symptoms, which results in bone loss, hampering the results of revision surgery. In a study with a mean follow-up of 7.5 years, Dalemans et al. [5] reported a drop in survival 6 years after surgery due to instability, infection and metallosis, but they did not see loosening of the components in their patients. The aim of this study was to assess the long-term results of the iBP elbow prosthesis.

## Methods

Between April 2000 and June 2009 37 primary iBP total elbow arthroplasties in 31 patients have been performed at University Medical Center of Groningen by 4 different orthopedic surgeons. All 31 patients (37 elbows) with a primary iBP were included in this study. Nineteen elbows were left and 18 were right elbows. Mean age of the patients was 55 at the time of surgery. Ten patients were male, 21 female. Indications for surgery were painful destruction of the elbow joint due to rheumatoid arthritis in 23 patients (28 elbows), posttraumatic osteoarthritis in 3 patients and haemophilic arthropathy in 5 patients (6 elbows). Patients’ characteristics are shown in Table 1.

**Table 1:**
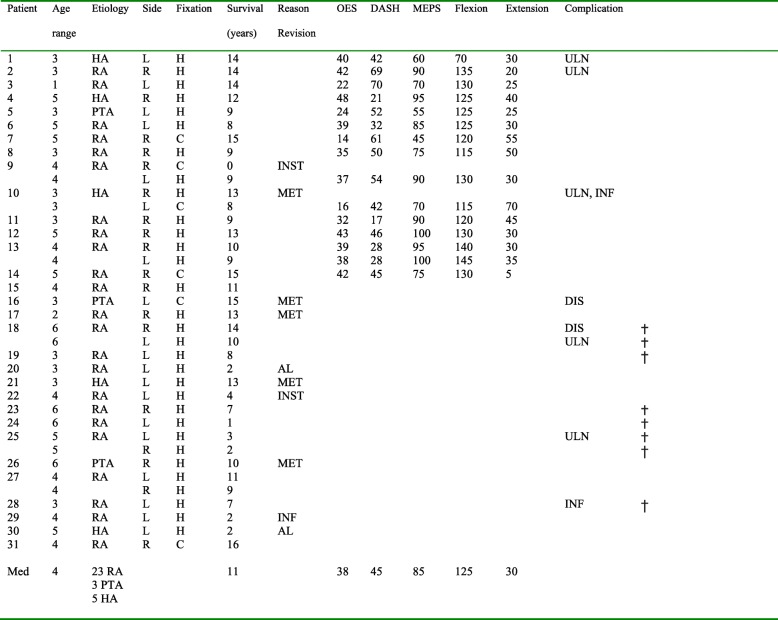
Patient characteristics

Age range (years): 1 = 20–29, 2 = 30–39, 3 = 40–49, 4 = 50–59, 5 = 60–69, 6 = 70–79,

*HA* haemophilic arthropathy, *RA* rheumatoid arthritis, *PTA* post-traumatic arthritis, *C* cemented, *H* hybrid (humeral component cementless), *AL* aseptic loosening, *INF* infection, *INST* instability, *MET* metallosis, *ULN* ulnaropathy, *DIS* = dislocation, † = died

The study was reviewed and approved by the Medical Ethical Committee of UMCG (METc2016/038). None of the surgeons was involved in the design of the implant.

### Surgical technique

In all cases a posterior approach was used with a triceps split technique as described by Pooley [3, 6]. The ulnar nerve was released and protected during the procedure. Release of the collateral ligaments were performed in case of contractures. The radial head was excised in all cases. The humerus and ulna were prepared with preservation of as much bone as possible according to the philosophy of this prosthesis. All humeral components were inserted without cement, except for 6 elbow prostheses in which poor bone quality urged to the use of cement. All ulnar components were cemented.

Post-operatively the elbow was protected by a removable cast for 4 weeks, avoiding active extension. Thereafter, the elbow was mobilized without brace and active triceps training was allowed. Patients were advised to limit weight bearing up to 1 kg repetitively and to 5 kg incidentally.

### Outcome measures

**Fig. 2:**
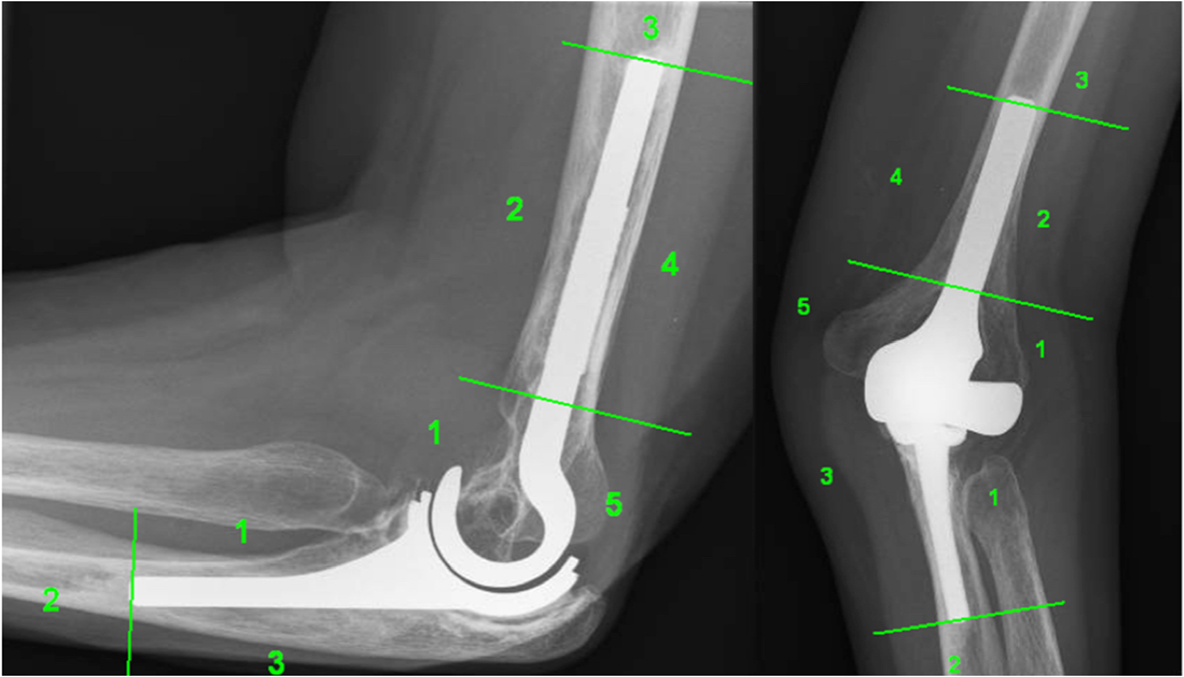
Classification of radiological analysis as described by Wagener et al. In each zone we looked for the presence of radiolucencies

To assess pain, elbow function and social-psychological status, the Oxford Elbow Score (OES) [7] was used. Disabilities of Arm, Shoulder and Hand Outcome Measure (DASH) [8] was used to assess upper-limb function. The Mayo Elbow Performance Score (MEPS) [9] was used to assess pain, range of motion and stability. Health-related quality of life was determined by using the EQ. 5D-3 L VAS score [10]. Pain at rest and during activities was scored from 0 (no pain) to 10 (severe pain) using visual analogue scales (VAS). Patients were also asked whether they were satisfied with their elbow function. By means of physical examination we determined the active range of motion (ROM). The integrity of the ulnar nerve was assessed using careful clinical examination. The stability of the medial and lateral collateral ligaments was scored as grade 0, no instability; grade 1, moderate instability (< 10°) and grade 2 severe instability (> 10°). All patients had standard anteroposterior (AP) and lateral radiographs of the elbow. Loosening of the implants was classified using the system described by Wagener et al. [11] (Fig. 2). Radiographs were also assessed of dislocation of the prosthesis, subluxation, periprosthetic fractures and signs of metallosis.

### Statistical analysis

SPSS statistical software (version 24.0, IBM SPSS, Chicago) was used. Descriptive statistics were used to describe patients’ characteristics, clinical outcomes and scores on the questionnaires. Kaplan-Meier survival analysis was performed with revision as an end point. Kruskal-Wallis Test for independent samples was used to analyze differences in indication for surgery.

## Results

**Fig. 3:**
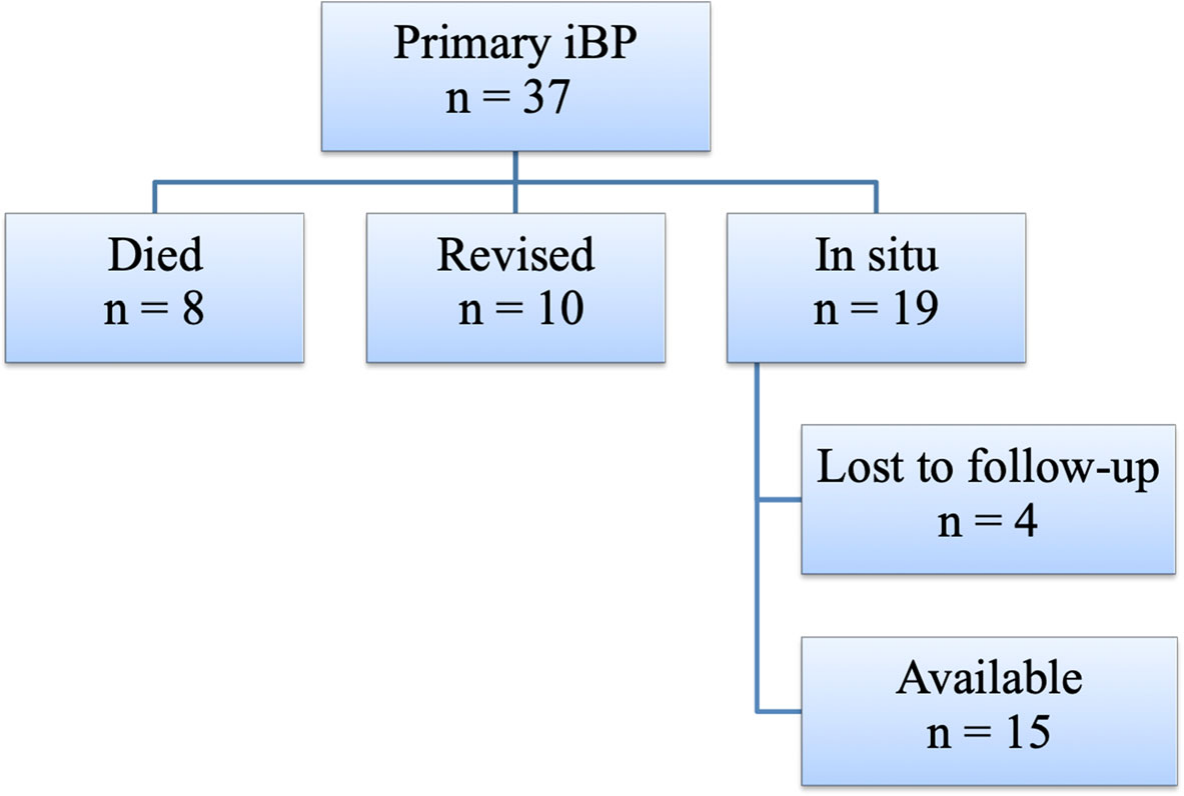
Flow diagram of iBP elbow prostheses

The mean follow-up of this study is 11 (8–15) years. At follow-up 6 patients (8 elbows) had died. Ten elbow prostheses in 10 patients had already been revised, leaving 19 primary prostheses in 17 patients in situ. Unfortunately, three patients (4 elbows) could not participate in our follow-up, as they were physically unable to come to the hospital. However, they did not have complaints of their elbow. Fourteen patients with 15 primary iBP elbow prostheses were available for clinical assessment (Fig. 3).

### Survival

**Fig. 4:**
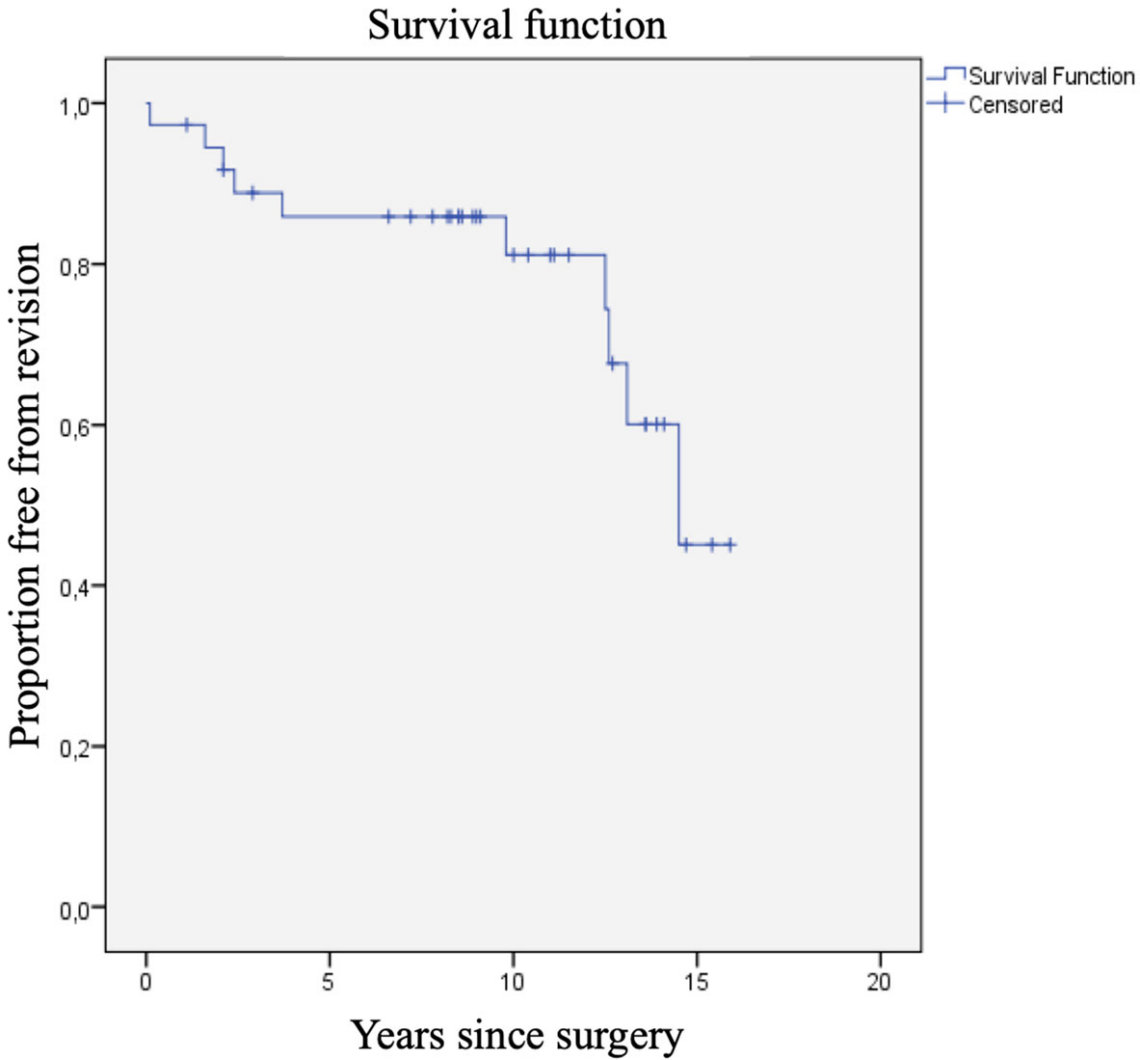
Kaplan-Meier survival analysis curve with revision for any reasons as an endpoint

**Fig. 5:**
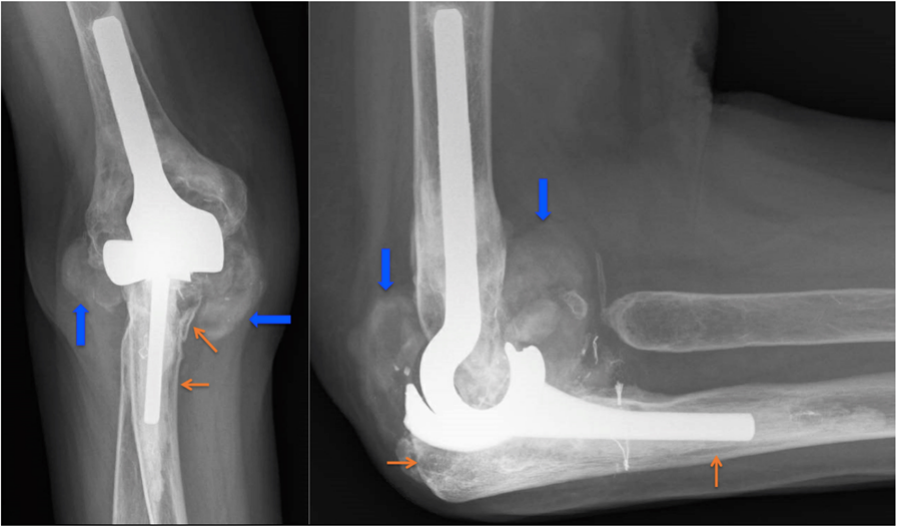
Radiological signs of PE wear and metallosis. Orange arrows showing radiolucencies, blue arrows showing pseudotumor

**Fig. 6:**
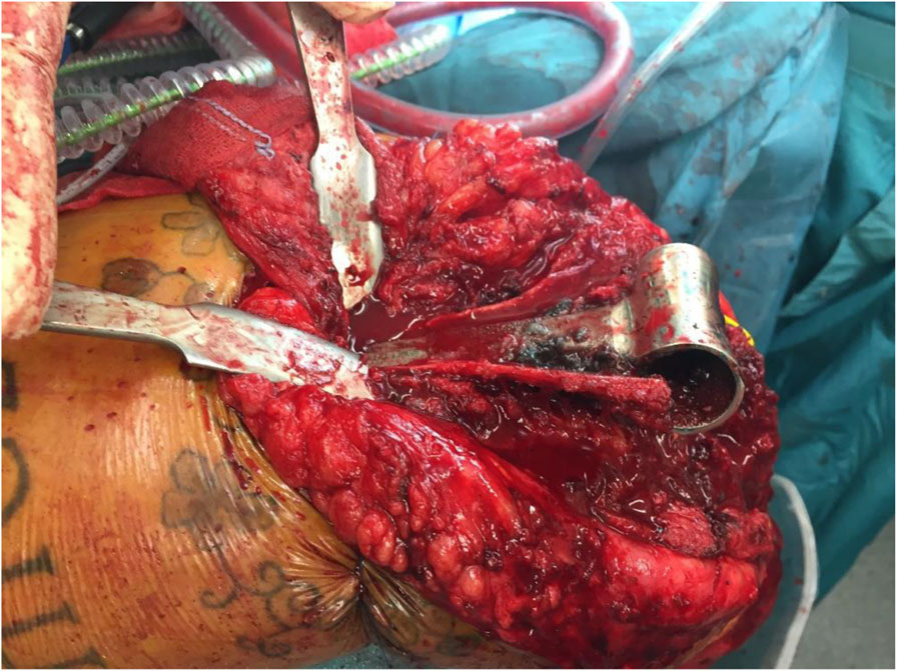
Revision arthroplasty by humeral osteotomy: severe metallosis

The Kaplan-Meier survival analysis (Fig. 4) showed survival rates of 88 and 81% respectively at 5 and 10 years after surgery. All 37 primary iBP’s were included in this analysis. Furthermore, a progressive decline in survival is visible beyond 10 years. Ten out of 37 iBP elbow prostheses had been revised. The reasons for early revision were infection in one patient, aseptic loosening in two patients and instability in two patients, which occurred in the first 4 years after surgery. The reason for late revision (after 9 years) was loosening with severe polyethylene wear and as a consequence metallosis in 5 patients (Figs. 5,6). Arrows show radiolucency (orange) around the prosthesis and severe pseudotumor (blue), indicating metallosis.

### Clinical assessment

Overall, most patients were satisfied with their iBP elbow prosthesis. Only 2 out of 14 patients were not satisfied with the result. One patient was not satisfied, because of ulnar neuropathy and loss of function. Another patient was no longer satisfied because of loosening of the ulnar component that had led to loss of function. No revision arthroplasty was planned though because of severe comorbidities. One patient was indifferent, which left 11 patients (12 elbows) being satisfied with their iBP elbow prosthesis.

Mean MEPS score was 80 points (45–100) indicating a fair-to-good result. Lower scores indicated pain in most cases. Mean DASH score was 44 points (17–70) and mean OES 34 points (14–48). This score usually indicates mild-to-moderate elbow complaints. The underlying disease, rheumatoid arthritis, mainly influenced both scores, as illustrated by the health-related quality of life score: 6 (4–8). Assessment of pain on a 0–10 point scale scored a mean of 2 (0–7), indicating low pain levels.

Mean ROM was 90° (40°-125°), mean flexion 125° (70°-145°), mean extension deficit 35° (5°-70°). Mean pronation 70° (40°-95°), mean supination 75° (40°-95°). Seven out of 15 elbows were unstable. Two of them were grossly unstable and 5 were moderately unstable, although this was not clinically relevant in terms of dislocation. Three of 14 patients had signs of persistent ulnar neuropathy. There were no significant differences in clinical outcomes and survival between indication for surgery.

### Radiological assessment

At the assessment, one out of 15 cases showed loosening of the ulnar component with a fracture in zone 3. Eleven out of 15 cases showed radiolucent lines, especially in zones 1 and 3 of the ulna. Three cases showed incongruity and another 3 cases showed signs of metallosis and pseudotumor (Table 2). Looking back at the already revised cases, 5 of 10 cases (all late revisions) had severe radiolucencies (especially zones 1 and 3 of the ulna) and signs of metallosis, which was confirmed at revision surgery.

**Table 2:**
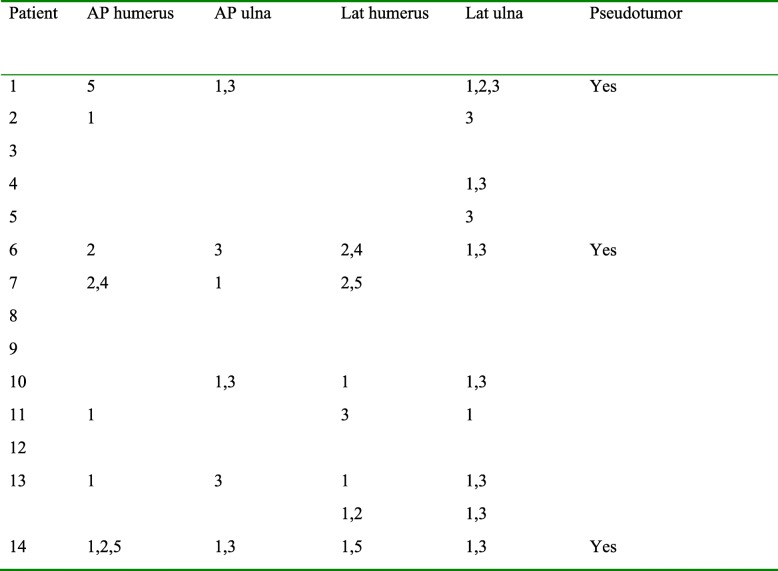
Radiological analysis

*AP* anteroposterior, *Lat* Lateral. Numbers indicate zone of radiolucency as described in Fig. 2

All together in 15 of 25 cases (revisions and survivals) the radiographs indicated bone loss due to particle disease with PE wear and metallosis as to be expected. The case shown in Fig. 5 demonstrated radiolucencies and pseudotumor, suggestive for metallosis. Painful loosening 10 years after primary surgery led to a revision in which severe bone loss was encountered.

## Discussion

The iBP elbow prosthesis is an unlinked prosthesis. The stability of the joint is provided by the soft tissues, therefore the risk of dislocation is higher than in a linked-type prosthesis [12, 13]. The theoretical advantage of this design is the lower risk of loosening, because of minimal stress on the bone-implant interface [5].

Our study shows survival rates for the iBP elbow prosthesis of 88 and 81% respectively at 5 and 10 years postoperatively with a progressive decline in survival beyond 10 years. Eleven of 14 patients were satisfied with their elbow as reflected in the scores of the questionnaires. In our study the main reason for revision was loosening with polyethylene wear and metallosis (50%), which is higher compared to the results of Dalemans et al.(33%), but they only had a follow-up of 7.5 years with their series of the iBP elbow prosthesis [5, 12, 13]. In their series with the Kudo 5, an unlinked prosthesis with a mean follow-up of 14.5 years, the main reason for revision was loosening and metallosis as well (61%). The unlinked prostheses were originally developed to render less loosening than linked prostheses, but Voloshin et al. showed in their systematic review no significant difference in clinically relevant loosening between linked and unlinked elbow prostheses [12].

Over the last years improvements in the quality of PE have been made. A recent study by Popoola et al. [14] showed that vitamin E blended and crosslinked polyethylene gives lower in vitro wear compared to conventional gamma-irradiated polyethylene in two types of linked, semiconstrained total elbow prostheses. Probably motion in combination with the thin polyethylene in the design of the iBP prosthesis eventually leads to wear, metallosis and loosening.

When revising the iBP we had to revise the ulnar component in 3 of 10 cases.

In 6 of 10 cases persistent instability due to debridement of soft tissue and insufficiency of the collateral ligaments, required revision to a linked-type total elbow prosthesis. In one case a complete iBP revision was performed.

The relatively short survival and the described wear problems and loosening of the ulnar component in combination with the difficulties experienced in revision of an even well-fixed humeral stem have made us decide to stop using this prosthesis.

Another well-known complication after TEA is ulnar neuropathy [15–18]. In our study 3 patients (20%) had some form of permanent ulnaropathy, despite the ulnar nerve was mobilized and protected during surgery. The reported outcomes on neuropathy after elbow prosthetic joint placement are conflicting. Tanaka et al. did not find ulnaropathy after release of the nerve [18]. On the other hand, not mobilizing the ulnar nerve as shown by Kleinlugtenbelt et al. resulted in 6% ulnaropathy [4], by Brinkman et al. a high rate of 20% ulnar neuropathy was found [15]. Postoperative ulnar neuropathy rates appear to vary, so we recommend further research to determine whether mobilization of the ulnar nerve is preferred.

Mean arc of motion in our study was 90°, with a mean flexion of 125° and a mean extension deficit of 35°. Mean pronation was 70° and mean supination 75°. Morrey et al. [19] described that the functional arc of motion required for activities in daily life was 120/30 for flexion/extension and 50/50° for pronation/supination. Five out of 14 patients had a range of motion inferior to this standard. Most of the patients were nonetheless satisfied with their elbow function.

Few studies have been conducted to determine the long-term outcome of the iBP elbow prosthesis. One study by Kleinlugtenbelt et al. [4] showed a discrepancy between clinical evaluation and radiological signs at short-term follow-up, with a mean follow-up of 4 years. Our study showed this discrepancy too.

Loosening of the ulnar component was seen in 11 patients, although 8 of them did not have any complaints of their elbow. In 9 of these 11 patients radiolucencies around the humeral component were seen. Seven patients had some degree of elbow instability at physical examination without dislocation; 3 of them did show radiological signs of subluxation.

Our current ‘care as usual’ does not routinely include medium or long term follow-up. The outcome of this study has changed our policy and we routinely do a structural follow-up. Patient related outcome measures only will not be sufficient to assess the integrity of these implants. Following this study, another three prostheses have been revised. All three of them with severe polyethylene wear and metallosis.

### Limitations

We were only able to examine a small number of patients. Our study had a retrospective design, which entailed an important loss of patients. The strength of this study is the length of follow-up.

## Conclusion

We warrant a structural follow-up for all iBP implants, because of the discrepancy between clinical signs and symptoms and radiological loosening, metallosis and consequent progressive bone loss.

## Data Availability

The datasets used and/or analyzed are available from the corresponding author on reasonable request.

## Abbreviations

iBP: instrumented Bone Preserving
OES: Oxford Elbow Score
DASH: Disabilities of Arm, Shoulder and Hand Outcome Measure
MEPS: Mayo Elbow Performance Score
TEA: Total elbow arthroplasty
VAS: Visual analogue scales
PE: Polyethylene
